# Echocardiographic markers of dyssynchrony as predictors of super-response to cardiac resynchronisation therapy – a pilot study

**DOI:** 10.1186/s12947-018-0140-0

**Published:** 2018-10-02

**Authors:** V. A. Kuznetsov, A. M. Soldatova, J. D. Kasprzak, D. V. Krinochkin, N. N. Melnikov

**Affiliations:** 1 Tyumen Cardiology Research Center, Tomsk National Research Medical Center, Russian Academy of Science, Tomsk, Russia, Melnikaite st. 111, Tyumen, 625026 Russia; 2 Department of Cardiology, Medical University of Lodz, Bieganski Hospital, Kniaziewicza 1/5, 91-347, Lodz, Poland

**Keywords:** Dyssynchrony, Cardiac resynchronisation therapy, Super-response

## Abstract

**Background:**

Some patients with congestive heart failure have greater improvement of cardiac remodelling after cardiac resynchronisation therapy (CRT) and they are identified as super-responders (SRs). It remains unclear if echocardiographic markers of dyssynchrony could accuratelly predict super-response to CRT. The aim of this study is to evaluate potential echocardiographic predictors associated with super-response to CRT.

**Methods:**

Fifthy nine CRT patients (mean age 52.9 ± 9.0 years, 88% men) with congestive heart failure (54% ischaemic and 46% non-ischaemic aetiology) II-IV NYHA functional class were enrolled. To assess mechanical dyssynchrony we evaluated interventricular mechanical delay, the maximum delay between peak systolic velocities of the septal and posterior walls of left ventricle, duration of left ventricular pre-ejection period (LVPEP), left ventricular and interventricular dyssynchrony by tissue Doppler imaging and systolic dyssynchrony index by 3D echocardiography. After six months the patients were assessed for response and classified as SRs (reduction in left ventricular end-systolic volume (LVESV) ≥30%, *n* = 20) and non-SRs (reduction in LVESV < 30%, *n* = 39) and baseline data were analyzed to identify the predictors.

**Results:**

Both groups demonstrated significant improvement in NYHA functional class, increase in left ventricular ejection fraction and reduction in LVESV. All parameters of mechanical dyssynchrony at baseline were significantly higher in SR group. Multiple logistic regression analysis showed that LVPEP (HR 1.031; 95% CI 1.007–1.055; *p* = 0.011) was an independent predictor for CRT super-response. In ROC curve analysis LVPEP with a cut-off value of 147 ms demonstrated 73.7% sensitivity and 75% specificity (AUC = 0.753; *p* = 0.002) for the prediction of super-response to CRT.

**Conclusion:**

Greater mechanical dyssynchrony is associated with super-response to CRT in patients with congestive heart failure. It is probable that an LVPEP > 147 ms can be used as independent predictor of super-response.

## Background

Cardiac resynchronisation therapy (CRT) is an effective treatment for patients with congestive heart failure with reduced ejection fraction (HF-rEF) and prolonged QRS duration. Several large multicentre clinical trials have confirmed that CRT can improve heart function, exercise capacity and quality of life. CRT reduces mortality and hospitalization and can also improve the prognosis in patients with HF-rEF [1]. This benefit is believed to result from the elimination of mechanical asynchrony of the heart.

A decrease in the left ventricular end-systolic volume (LVESV) ≥15% is used as a standardized criterion of CRT response. However, some patients show greater improvement in cardiac function after CRT and are identified as super-responders (SRs) [2].

## Aim

The aim of the study was to evaluate potential echocardiographic predictors associated with super-response to CRT.

## Methods

This study enrolled 59 patients from a local database of implanted CRT devices (mean age 52.9 ± 9.0 years, 88% men) with HF-rEF (32 patients with ischaemic and 27 with non-ischaemic aetiology) [3]. Patients were enrolled where there were available full echocardiographic data at baseline and in terms of dynamics (two-dimensional (2D) and three-dimensional (3D) echocardiography). In 41 patients, we implanted combined devices with a defibrillator function (CRT-D).

The main criteria for CRT implantation were: New York heart association (NYHA) functional class II-IV, reduced left ventricular ejection fraction (LVEF) < 35%, interventricular and/or intraventricular dyssynchrony assessed by echocardiography, with QRS width taken into account [1, 4]. All patients received medical treatment in accordance with the current guidelines. Device implantation was effective in all patients and occurred without complications.

According to the change in LVESV assessed after six months, the patients were divided into two groups: I) SR (decrease ≥30%; *n* = 20); II) non–SR (decrease < 30%, *n* = 39) [5]. Clinical characteristics of the study participants are shown in Table 1.

**Table 1:** Baseline characteristics of the study groups

Parameter	*N* = 59	I group (*n* = 20)	II group (*n* = 39)	*Р*
Age, (years)	52.9 ± 9.0	52.0±7.6	53.4±9.7	0.624
Men, (n)	88% (52/59)	85% (17/20)	90% (35/39)	0.594
CAD, (%)	54	47	60	0.308
NYHA functional class	2.8±0.6	2.6±0.6	2.8±0.6	0.110
LBBB, (%)	59	50	64	0.297
QRS, (ms)	140.9 ± 38.9	139.1±44.2	141.8±36.7	0.997
Atrial fibrillation, (%)	34	25	38	0.301
LVEDV, (ml)	231.9±65.1	233.4±65.0	231.2±65.9	0.288
LVESV, (ml)	163.5±49.4	163.4±46.5	163.5±51.3	0.475
LVEF, (%)	29.8±3.6	30.0±2.7	29.6±3.9	0.458

Tables. 1 and 2: *M* ± *SD* - mean ± standard deviation, *CAD* – coronary artery disease, *NYHA* – New York Heart Association, *LBBB*- left bundle branch block, *LVESV* – left ventricular end-systolic volume, *LVEDV* – left ventricular end-diastolic volume, *LVEF* – left ventricular ejection fraction

Standard echocardiography was performed using a commercially available system, i.e., Philips IE 33. Patients underwent baseline and six-month post-implantation echocardiography. The examination included 2D grayscale, colour and spectral blood pool Doppler and tissue Doppler imaging (TDI). Three cardiac cycles were obtained for each acquisition. 3D echocardiograpms were recorded as multibeat electrocardiography-gated data sets, which were obtained over one cardiac cycle. Using 3D echocardiography, we evaluated the left ventricular end-diastolic volume (LVEDV), LVESV, LVEF, stroke volume and systolic dyssynchrony index (SDI) [6, 7]. All Doppler measurements were averaged from three beats.

As for dyssynchrony parameters, three mechanical dyssynchrony indexes were quantified by 2D echocardiography: septal-to-posterior wall motion delay (SPWMD) (> 130 ms), interventricular mechanical delay (IVMD) as the delay in the onset of outflow between the left and right ventricle (with abnormality cut-off> 40 ms), and left ventricular preejection period (LVPEP) (abnormality cut-off > 140 ms). Interventricular (abnormality cut-off > 120 ms) and intraventricular (abnormality cut-off > 60 ms) dyssynchrony were assessed by TDI based on the difference between time to onset of the systolic velocity spectrum, recorded from the lateral tricuspid and lateral mitral annulus, using four-chamber apical view (interventricular) and lateral and septal left ventricular annulus, using four-chamber apical view (intraventricular) asynchrony. Additionally, SDI ≥5.6% assessed by 3D echocardiography, was applied as a criterion of interventricular dyssynchrony [8].

Statistical analyses were performed using SPSS for Windows version 21.0 (SPSS Inc., Chicago, IL, USA). All values had normal distribution, with results expressed as mean value±standard deviation (mean ± SD), while mean changes in echocardiographic parameters were expressed as median and the interquartile range (Ме [25%; 75%]). Continuous variables were compared using Student’s *t* test. The χ^2^ or Fisher’s exact test was used to compare categorical variables. Differences in continuous variables between the baseline and follow-up visits were compared using paired *t*-tests. Multiple logistic regression analysis was used to evaluate potential predictors related to super-response. ROC-analysis was used to assess the sensitivity and specificity of evaluated parameters in predicting CRT super-response. *p* < 0.05 was considered to be statistically significant.

## Results

At baseline, there were no differences in the demographic, clinical and functional characteristics between the groups (Table 1). After six months, 20 out of 59 (34%) patients were classified as SRs.

Six months after implantation, both groups demonstrated a significant decrease in NYHA functional class, increase in 6-min walking distance and improvement in echocardiographic parameters according to 3D echocardiography. The improvement in these parameters was significantly higher in SRs, as defined by reverse remodeling (Table 2), including the larger decrease in LVEF.

**Table 2:** Clinical and functional characteristics at baseline and after 6 months of CRT

Parameter		I group (*n* = 20)	II group (*n* = 39)	*P*
NYHA functional class	At baseline	2.6±0.6	2.8±0.6	0.107
	After 6 months	1.9±0.7^a^	2.2±0.9^a^	0.169
6-min walking distance, (m)	At baseline	329.2±86.0	320.9±98.7	0.651
	After 6 months	421.5±66.7^a^	381.5±112.2^a^	0.169
LVEF by 3D echocardiography, (%)	At baseline	30.0±2.7	29.6±3.9	0.458
	After 6 months	40.1±5.4^a^	34.4±3.8^a^	< 0.001
	Mean changes	10[6.2;13.2]	5[3;7]	< 0.001
LVEDV by 3D echocardiography, (ml)	At baseline	233.4±65.0	231.2±65.9	0.820
	After 6 months	159.1±39.4^a^	213.2±63.7^a^	< 0.001
	Mean changes	−73.0[− 101.0;-51.7]	−15.0[− 31.0;-8.0]	< 0.001
LVESV by 3D echocardiography, (ml)	At baseline	163.4±46.5	163.5±51.3	0.957
	After 6 months	95.8±27.1^a^	140.0±46.7^a^	< 0.001
	Mean changes	−61.0[−91.0;-47.7]	− 20.0[− 38.0;-12.0]	< 0.001
QRS, (ms)	At baseline	139.1±44.2	141.8±36.7	0.996
	After 6 months	153.6±26.2	160.2±26.0^a^	0.367

^a^ – difference between baseline and postimplant value (*р* < 0.05)

At baseline, the parameters of mechanical dyssynchrony were significantly more pronounced in SRs: the values of SDI, LVPEP, IVMD, and interventricular delay, as assessed by TDI, were significantly higher in this group (Table 3). After six months, the parameters of mechanical dyssynchrony decreased significantly in both groups (Table 3). As mean changes of dyssynchrony parameters were more pronounced in SR group in terms of dynamics, the mean values of these parameters did not differ between groups (Table 3).

**Table 3:** Parameters of mechanical dyssynchrony at baseline and after 6 months of CRT

Parameter		I group (*n* = 20)	II group (*n* = 39)	*Р*
Systolic dyssynchrony index, (%)	At baseline	9.5±3.4	7.5±4.4	0.05
	After 6 months	2.8±1.2^a^	3.1±1.2^a^	0.278
	Mean changes	−6.4[−8.7;-4.3]	−2.8[− 8.2;-0.36]	0.044
Left ventricular pre-ejection period, (ms)	At baseline	160.5±31.2	131.5±29.5	0.002
	After 6 months	127.7±22.8^a^	118.3±21.2^a^	0.292
	Mean changes	−35.5[−53.7;-11.2]	−18.5[− 35.0;1.7]	0.043
Right ventricular pre-ejection period, (ms)	At baseline	102.1±21.4	95.4±18.6	0.249
	After 6 months	106.4±16.7	103.3±26.2	0.716
	Mean changes	9.5[−17.0;30.7]	14.5[−13.5;33.5]	0.512
Interventricular mechanical delay, (ms)	At baseline	57.5±28.3	39.7±24.2	0.024
	After 6 months	19.9±15.9^a^	25.4±11.7	0.357
	Mean changes	−47.0[−55.0;-13.0]	−11.0[−36.5;3.0]	0.054
Interventricular delay by TDI, (ms)	At baseline	110.2±66.1	71.6±57.9	0.038
	After 6 months	58.4±56.7^a^	50.6±48.3^a^	0.653
	Mean changes	−31.0[−120;12.5]	−20.5[−66.2;8.2]	0.165
Intraventricular delay by TDI, (ms)	At baseline	88.4±45.3	67.7±42.8	0.098
	After 6 months	54.7±44.9^a^	36.9±25.5	0.568
	Mean changes	−57.5[−80.2;-28.0]	−12.0[− 40.0;15.0]	0.005

*TDI* – tissue Doppler imaging

^a^ – difference between baseline and postimplant level (*р* < 0.05)

**Fig. 1:**
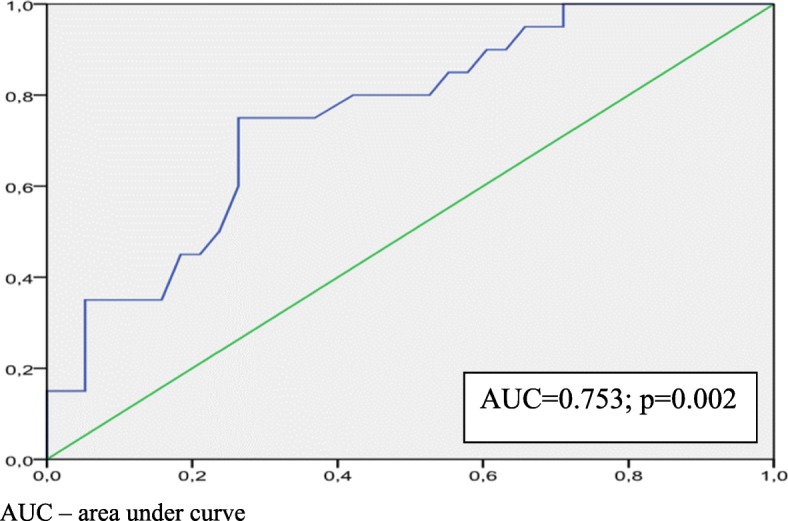
ROC-curve for sensitivity and specificity of LVPEP in prediction of response to CRT. AUC – area under curve

Multiple logistic regression analysis showed that only LVPEP (HR 1.031; 95% CI 1.007–1.055; *p* = 0.011) was an independent predictor for CRT super-response. In ROC curve analysis, LVPEP with a cut-off value of 147 ms demonstrated 73.7% sensitivity and 75% specificity (AUC = 0.753; *p* = 0.002) for the prediction of super-response to CRT (Fig. 1).

## Discussion

CRT in an effective treatment modality for patients with significant HF-rEF; however, approximately one third of patients do not benefit from this therapy [9]. In contrast, some patients show greater improvement in the cardiac function after CRT implantation and are identified as SRs; this is linked to improved clinical outcomes [10, 11], although there is no allowance for the deactivation of CRT as the ventricular dysfunction will return [10]. Our study has identified a simple, Doppler based and thus potentially robust criterion predictive of LV superresponse – LVPEP.

Super-response to CRT was first described in two studies. Blanc et al. found that, in a group of patients with non-ischaemic cardiomyopathy and a left bundle branch block (LBBB), some exhibited a normalization of LVEF *>* 50% after CRT [12]. Bulava et al., meanwhile, described a case study of a patient suffering from severe HF-rEF. The case represented exceptional left ventricular reverse remodelling with practically normalized left ventricular function after one year of synchronized pacing [13]. In recent studies, the dynamics of LVEF were used as a criterion of super-response; however, their level differed from study to study [14, 15].

To date, there is no universally accepted criterion of super-response. Some authors defined SRs in terms of an improvement in LVESV although different cut-offs were selected [16, 17]. In our study, a super-response was defined as a relative reduction in LVESV > 30% after six months of CRT according to several studies [2, 11, 12]. Steffel et al. previously compared three definitions for a super-response: defined by an absolute increase in LVEF of 10%, a decrease in the LVESV index by 30% and a decrease in the LVEDV index by 20%. The authors found that any of these cut-off points is highly predictive of clinical improvement and survival after CRT implantation [16]. A large study by Ypenburg linked the definition, which was accepted for this study (reduction in LVESV ≥30%), with improved clinical outcomes [5].

Prior clinical studies have reported the incidence of super-response to be in the range of 10–29% [18]. In the majority of studies, super-response was defined as LVEF > 50% together with a functional recovery NYHA class of I or II [19, 20]. In other studies, a super-response was defined as decrease in LVESV ≥30% [5, 21]. At the six-month follow-up, stage 22% of patients were defined as SRs (32% without LBBB). SR presented more extensive mechanical dyssynchrony at baseline [5]. In the case of PROSPECT sub-analysis, the percentage of SRs at six-months follow-up was 37.8%, they also presented more evidence of mechanical dyssynchrony [21]. In our study, we observed a high percentage of SRs (34%), although this corresponded to other studies in which the same SR criteria were used. The lack of a universal definition of super-response to CRT is presumably one of the main reasons for such a wide discrepancies between studies. The positive results from our study could be explained by good patient selection, along with the decision to assess mechanical dyssynchrony parameters using 2D and 3D echocardiography. The utilization of mechanical dyssynchrony parameters for patient selection to CRT may also explain the significant positive effect of CRT in the CARE-HF trial [4].

Different factors associated with super-response to CRT have been reported. Jin reported that biventricular pacing percentage greater than 98% was a good predictor of a super-response [22]. Some data suggest that patients with non-ischemic cardiomyopathy and an absence of myocardial infarction receive the greatest benefit from CRT, while patients with ischaemic heart disease tend to respond to CRT to a less extent than patients with non-ischaemic cardiomyopathy [14, 16, 23]. According to PROSPECT sub-analysis, patients with ischaemic HF showed less improvement in LVESV after CRT, although it is noteworthy that 31.8% of them were classified as SRs [21]. In our study, 54% of patients (*n* = 32) had ischaemic aetiology of HF, while 71.9% of them (*n* = 23) were SRs. In univariate and multivariate analysis non-ischaemic aetiology of HF was not associated with super-response to CRT.

In our study, SRs demonstrated more pronounced improvement in echocardiographic parameters and an increase in LVEF. At the same time, it is obvious that the group of non-SRs (*n* = 39) was not homogeneous: 41% of patients (*n* = 16) were non-responders (a decrease in LVESV < 15%) and 59% of patients (n = 23) were responders (a decrease in LVESV 15–29%). Furthermore, the effect of CRT could be quite different between these subgroups, but this issue needs further investigation.

Despite different response criteria, in most studies with LBBB derived greater benefits from CRT [1]. Large multicentre studies have demonstrated that LBBB is associated with CRT response and long-term survival. At the same time, patients with non-LBBB morphology of QRS are less representative in clinical trials (less than 15%); therefore, little can be definitively inferred regarding the efficacy of CRT among these patients. There is also a lack of standardization in terms of QRS width and morphology in clinical trials and large studies [24]. Thus, in real clinical practice, the significance of QRS morphology for patient selection to CRT is still not clear. The presence of left hemiblock in right bundle branch block patients improved outcomes and, according to Rocha et al., these patients also became SRs (46% of the total SRs) [23, 25]. The success of CRT in patients with non-LBBB morphology of QRS can be associated with the presence of mechanical dyssynchrony [26, 27]. In our study LBBB was observed in 50% of SRs and 64% of non-SRs. Both groups demonstrated significant improvement in NYHA functional class, a decrease in LVESV and LVEDV and an increase in LVEF. However, improvement in these parameters was significantly higher in SRs. LBBB was not found to be a predictive factor of a greater response to CRT.

There is a link between electrical and mechanical dyssynchrony, but it is not always clearly identified. Some patients with normal QRS demonstrate significant mechanical disorders, however the question about the utilization of mechanical dyssynchrony parameters to predict CRT response is still unanswered [6, 7]. Some authors have described TDI and speckle tracking echocardiography parameters as predictors of CRT response [28–30]. In our study, the groups did not differ in terms of QRS width, while the mean level of QRS in SRs and non-SRs was relatively low (139.1±44.2 ms and 141.8±36.7 ms, respectively) but the parameters of mechanical dyssynchrony significantly differed between groups, with greater mechanical dyssynchrony in patients with super-response to CRT. Among the SRs, all parameters of inter- and intraventricular dyssynchrony decreased after six months of CRT; among the non-SRs, the parameters of intraventricular dyssynchrony also reduced significantly. However, the width of the QRS complex increased in both groups. This may be explained by the widening of the QRS complex in patients with narrow baseline QRS (< 120 ms), given that CRT conduction differs from normal conduction, such that we can create artificial electrical dyssynchrony in these patients. At the same time, a significant clinical effect and reverse remodelling in SRs support the idea of a positive effect of CRT on the elimination of mechanical dyssynchrony, which is more significant than in the appearance of electrical disorders.

### Limitations

Our study has several limitations. This pilot study only involved a single centre and a retrospective protocol. The patient count was also limited; however, we were able to identify a number of SRs that was sufficient for statistical comparison purposes.

The study enrolled patients from a local database of implanted CRT devices; thus, the heterogeneity of patients included in this study was the result of real clinical practice. 54% of patients enrolled on our study had ischaemic aetiology of HF, which, in percentage terms, corresponds to a study cohort in large multicentre studies [31, 32].

The mean QRS width in our patients was 140.9 ± 38.9 ms, while 59% of patients had LBBB. Enrolment began in January 2009 and ended in December 2015. Until 2012 (about half of the enrolment period), a QRS width > 120 ms was one of the main criteria for CRT implantation. We enrolled patients with a QRS width > 120 ms or <120 ms + two parameters of mechanical dyssynchrony. It should be noted that, from 2005 until the latest update to the clinical recommendations in 2013, in our clinic, we used the St. Mary’s Hospital and Imperial College (London) protocol for CRT implantation, which included parameters of mechanical dyssynchrony assessed by TDI [33].

New echocardiographic techniques, such as speckle tracking, visual criteria of dyssynchrony as apical rocking, septal flash and visual late lateral activation, were not evaluated.

Due to our retrospective design, no intra- or interobserver reproducibility analysis of this study was performed regarding electrocardiographic and echocardiographic variables.

## Conclusion

Greater mechanical dyssynchrony is associated with super-response to CRT. It is probable that an LVPEP > 147 ms can be used as an independent predictor of super-response.

## Abbreviations

2D: 2-dimensional
3D: 3-dimensional
CRT: Cardiac resynchronisation therapy
CRT-D: Cardiac resynchronisation therapy with defibrillator function
HF-rEF: Heart failure with reduced ejection fraction
IVMD: Interventricular mechanical delay
LBBB: Left bundle branch block
LVEDV: Left ventricular end-diastolic volume
LVEF: Left ventricular ejection fraction
LVESV: Left ventricular end-systolic volume
LVPEP: Left ventricular preejection period
NYHA: New York heart association
SDI: Systolic dyssynchrony index
SPWMD: Septal-to-posterior wall motion delay
SR: Super-responder
TDI: Tissue Doppler imaging
